# Differential cell-type-expression of CYFIP1 and CYFIP2 in the adult mouse hippocampus

**DOI:** 10.1080/19768354.2019.1696406

**Published:** 2019-11-24

**Authors:** Yinhua Zhang, Hyae Rim Kang, Kihoon Han

**Affiliations:** a Department of Neuroscience, College of Medicine, Korea University, Seoul, South Korea; b Department of Biomedical Sciences, College of Medicine, Korea University, Seoul, South Korea

**Keywords:** CYFIP1, CYFIP2, scRNAseq, cell type, hippocampus

## Abstract

Recent molecular genetic studies have suggested that two members of the cytoplasmic FMR1-interacting protein (*CYFIP*) gene family, *CYFIP1* and *CYFIP2*, are causally associated with several brain disorders. However, the clinical features of individuals with *CYFIP1* and *CYFIP2* variants are quite different. In addition, null mice for either *Cyfip1* or *Cyfip2* are lethal, indicating that these two genes cannot compensate for each other in vivo. Although these results strongly suggest that CYFIP1 and CYFIP2 have distinct functions in vivo, the detailed mechanisms underlying their differences remain enigmatic and unexplored, especially considering their high sequence homology. To address this, we analyzed a recently established mouse brain single-cell RNA sequencing (scRNAseq) database and found that *Cyfip1* and *Cyfip2* are dominantly expressed in non-neurons and neurons, respectively, in all tested brain regions. To validate these observations, we performed fluorescent immunohistochemistry in the adult mouse hippocampus with either a CYFIP1 or CYFIP2 antibody combined with antibodies for various cell-type-specific markers. Consistent with our analysis of the scRNAseq database, CYFIP1 signals were detected in both neurons and astrocytes, while CYFIP2 signals were mainly detected in neurons. These results suggest differential cell-type-expression of CYFIP1 and CYFIP2 in vivo, which provides novel insights into our understanding of the pathophysiology of and potential treatments for *CYFIP*-associated brain disorders.

## Main text

The cytoplasmic FMR1-interacting protein (CYFIP) family proteins (CYFIP1 and CYFIP2) are evolutionarily conserved, ∼145 kDa proteins that are involved in the regulation of messenger RNA (mRNA) translation and actin dynamics in the nervous system (Abekhoukh and Bardoni 2014; Zhang et al. 2019). CYFIP1 and CYFIP2 have high amino acid sequence homology (88% identity and 95% similarity), suggesting similar functions at the molecular level. Specifically, both proteins interact with fragile X mental retardation protein (FMRP), an mRNA-binding protein whose loss causes fragile X syndrome (Schenck et al. 2001). Moreover, either CYFIP1 or CYFIP2, together with four other proteins, form the heteropentameric WAVE regulatory complex (WRC), which is a critical regulator of cellular actin dynamics (Abekhoukh and Bardoni 2014; Lee et al. 2017).

Despite their similar molecular functions, several lines of evidence indicate that CYFIP1 and CYFIP2 have distinct functions in vivo. For example, both *Cyfip1*- and *Cyfip2*-null mice are lethal at different developmental time points (i.e. at early embryonic and perinatal stages, respectively) (Chung et al. 2015; Han et al. 2015; Zhang et al. 2018). More importantly, genetic variants of *CYFIP1* and *CYFIP2* are associated with different types of brain disorders. Deletions and duplications of the chromosomal region harboring *CYFIP1* (15q11–13) are associated with autism spectrum disorders, intellectual disability, and schizophrenia (Abekhoukh and Bardoni 2014; Bagni and Zukin 2019). In contrast, recent whole-exome and -genome sequencing studies identified de novo *CYFIP2* variants in individuals with early-onset epileptic encephalopathy, which is characterized by developmental delay and seizures (Nakashima et al. 2018; Peng et al. 2018; Zweier et al. 2019). However, the detailed mechanisms underlying the in vivo differences between CYFIP1 and CYFIP2 remain largely unexplored, which is an important issue toward understanding the pathophysiology of and potential treatments for various *CYFIP*-associated brain disorders.

**Figure 1.**
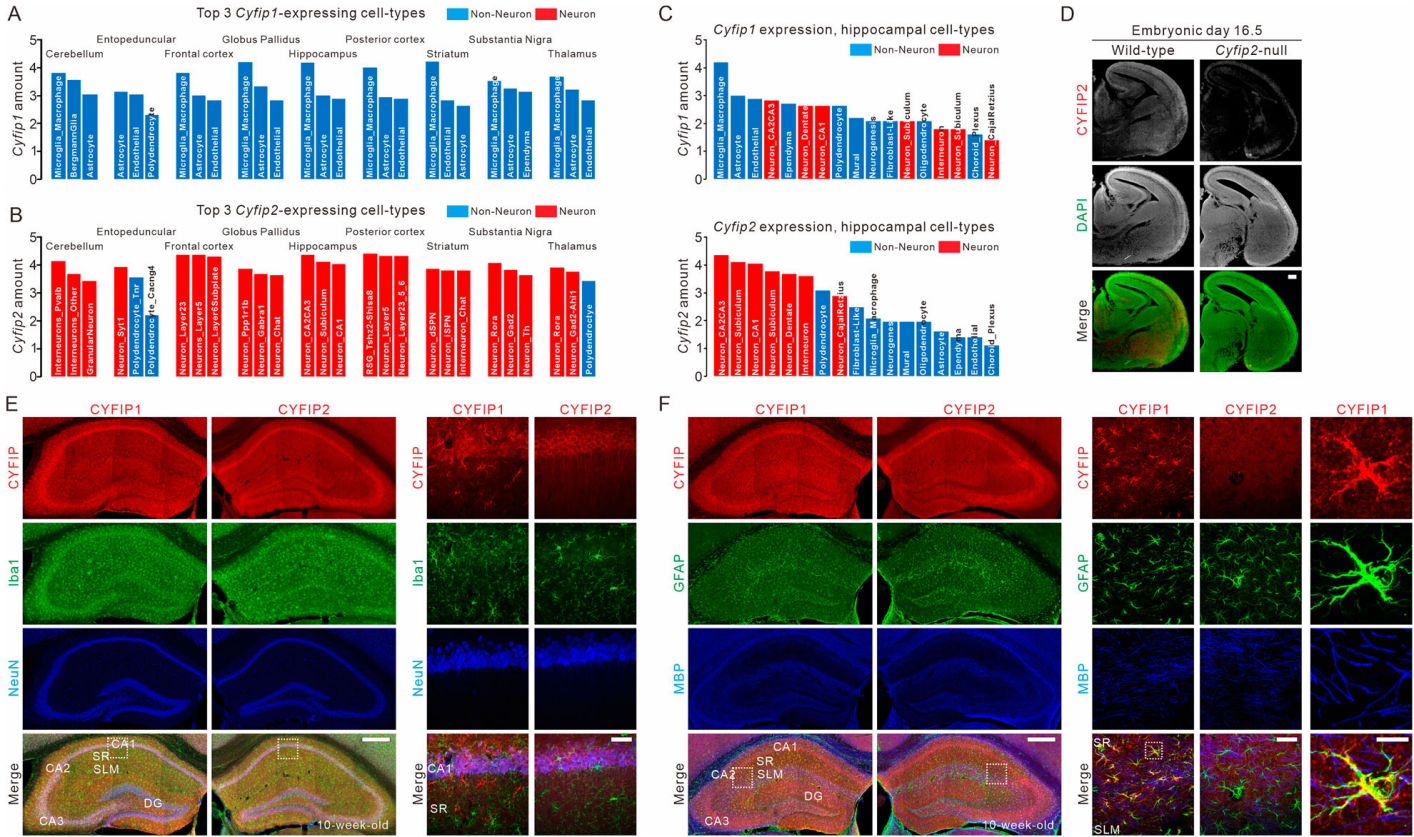
Differential cell-type-expression of CYFIP1 and CYFIP2 in the adult mouse hippocampus. (A) Bar graph showing the three cell types with the highest *Cyfip1* expression levels in nine different regions of the adult mouse brain. The values were obtained from the DropViz database (http://dropviz.org/). (B) Bar graph showing the three cell types with the highest *Cyfip2* expression levels in nine different regions of the adult mouse brain. Blue bar, non-neurons; red bar, neurons. (C) Bar graphs showing *Cyfip1* (upper panel) and *Cyfip2* (lower panel) expression levels across 17 different hippocampal cell types. (D) Confocal images of fluorescent immunohistochemistry (IHC) using CYFIP2 antibody in the brain sections from embryonic day 16.5 wild-type and *Cyfip2*-null mice. DAPI is a nuclear counterstain. Scale bar, 200 μm. (E) Confocal images of fluorescent IHC using CYFIP1, CYFIP2, Iba1, and NeuN antibodies in the adult mouse hippocampus. The right panels are high magnification images of the regions in dotted-line boxes in the left panels. DG, dentate gyrus; SR, stratum radiatum; SLM, stratum lacunosum and moleculare. Scale bars, 400 μm (left panel) and 40 μm (right panel). (F) Confocal images of fluorescent IHC using CYFIP1, CYFIP2, GFAP, and MBP antibodies in the adult mouse hippocampus. Scale bars, 400 μm (left panel), 40 μm (middle panel), and 10 μm (right panel).

The recent development of single-cell RNA sequencing (scRNAseq) technology has provided an unprecedented opportunity to characterize the cellular complexity of various organs, including the brain (Saunders et al. 2018). In addition to identifying and classifying cell types based on their molecular specializations, scRNAseq data can also be useful for determining the expression profiles of genes of interest across different cell types in specific organs. Therefore, we searched the *Cyfip1* and *Cyfip2* expression profiles in the DropViz database (http://dropviz.org/), which was generated by scRNAseq analysis of 690,000 individual cells from nine different regions of the adult mouse brain (Saunders et al. 2018). Unexpectedly, we found a marked contrast between the expression profiles of *Cyfip1* and *Cyfip2*. Specifically, in the nine different brain regions, *Cyfip1* expression levels were higher in non-neuronal cells than in neurons (Figure 1(A)). In most brain regions, microglia, astrocytes, and endothelial cells were the three cell types with the highest *Cyfip1* expression levels. In contrast, *Cyfip2* was more abundantly expressed in neurons than in non-neuronal cells, in all tested brain regions (Figure 1(B)). Based on these intriguing findings, we further validated the cell-type-specific expression of CYFIP1 and CYFIP2 proteins by fluorescent immunohistochemistry (IHC) analysis of the mouse hippocampus (Figure 1(C)).

Both CYFIP1- and CYFIP2-specific antibodies are commercially available, but only the CYFIP1 antibody has been validated for IHC (Yoon et al. 2014). Therefore, we first validated the CYFIP2 antibody for IHC using brain sections from embryonic day 16.5 (E16.5) *Cyfip2*-null mice and their wild-type littermates (Figure 1(D)). We used embryonic brains because *Cyfip2*-null mice die soon after birth (Zhang et al. 2018). Then, we performed IHC in the hippocampus of adult (10-week-old) wild-type mice, with either the CYFIP1 or CYFIP2 antibody combined with antibodies against four cell-type-specific markers (i.e. neuronal nuclei [NeuN] for neuron, glial fibrillary acidic protein [GFAP] for astrocytes, ionized calcium-binding adapter molecule 1 [Iba1] for microglia, and myelin basic protein [MBP] for oligodendrocytes). We detected signals for both CYFIP1 and CYFIP2 in the NeuN-positive neurons of the CA1–3 and dentate gyrus (DG) subregions of the hippocampus (Figure 1(E)). Notably, discrete clusters of CYFIP1 signals were also observed in the dendritic layers (i.e. stratum radiatum, lacunosum, and moleculare) of the hippocampus, locations where the CYFIP2 signals were diffuse. Comparisons with cell-type-specific markers revealed that the discrete CYFIP1 signals were highly co-localized with GFAP-positive astrocytes (Figure 1(F)). Indeed, 89% of the CYFIP1 clusters in the dendritic layers were positive for GFAP, and 78% of the GFAP clusters in the dendritic layers were positive for CYFIP1. However, neither CYFIP1 nor CYFIP2 was significantly co-localized with Iba1 or MBP (Figure 1(E,F)).

Taken together, these results suggest differential cell-type-expression of CYFIP1 and CYFIP2 in the adult mouse hippocampus, with CYFIP1 expression in neurons and astrocytes, and CYFIP2 expression mainly in neurons. Notably, previous reports showed distinct morphological and functional phenotypes in the hippocampus of *Cyfip1* and *Cyfip2* heterozygous mice (Bozdagi et al. 2012; Han et al. 2015). Whether differential cell-type-expression of CYFIP1 and CYFIP2 contributes to these phenotypic differences is an interesting topic for future study. More broadly, we believe that the approach used in this study can be applied to other gene families, which may provide novel insights toward understanding gene family member-specific expression and function in vivo.

## Materials and methods

### Mice

The *Cyfip2*-mutant mice used in this study have been described previously (Han et al. 2015; Zhang et al. 2018). The wild-type and *Cyfip2* mice were bred and maintained on a C57BL/6J background, and all mice used in experiments were obtained by heterozygous mating (*Cyfip2^+/-^* X *Cyfip2^+/-^*) according to the Korea University College of Medicine Research Requirements. All procedures were approved by the Committees on Animal Research at Korea University College of Medicine (KOREA-2016-0066). The mice were fed *ad libitum* and housed under a 12 h light–dark cycle.

### Immunohistochemistry

For embryonic brains, pregnant female mice were deeply anesthetized with isoflurane and sacrificed. The brains of embryonic day 16.5 (E16.5) mice were extracted and fixed with 4% paraformaldehyde (PFA) in phosphate-buffered saline (PBS) three overnight. After fixation, brains were washed with PBS and cryoprotected with 30% sucrose in PBS for 48 h. Frozen brains in O.C.T compound (SAKURA Tissue-Tek, 4583) were sectioned (100 μm) using a cryostat microtome (Leica, CM3050S). For adult brains, mice were anesthetized with isoflurane and transcardially perfused with heparinized (20 units/mL) PBS followed by 4% PFA in PBS. Brains were extracted and post-fixed in 4% PFA overnight. After post-fixation, the brains were washed with PBS and cryoprotected with 30% sucrose in PBS for 48 h. Brains were frozen in O.C.T compound and sectioned (60 µm) using a cryostat microtome. The following primary antibodies were used: CYFIP1 (Millipore, AB6046), CYFIP2 (Abcam, ab95969), NeuN (Millipore, MAB377), Iba1 (Synaptic System, 234–006), MBP (BioLegend, 808401), and GFAP (Abcam, ab4674). DAPI (DAPI dilactate, Invitrogen, 300 nM in PBS) was used to counterstain nuclei. Confocal microscopy (Zeiss, LSM800) was used for image acquisition. Whole hippocampal regions were obtained by tile scanning and each frame was taken by Z-stacks of slices. Tiled Z-project images were aligned and turned into a single flattened image using ZEN software (Zeiss).
